# Development and validation of a secondary school classroom engagement instrument in math and science in the Ethiopian context

**DOI:** 10.3389/fpsyg.2025.1491615

**Published:** 2025-02-17

**Authors:** Alemayehu Berhanu, Tesfaye Semela, Belay Moges

**Affiliations:** ^1^School of Teacher Education, College of Education, Hawassa University, Hawassa, Ethiopia; ^2^Department of Psychology, Dilla University, Dilla, Ethiopia; ^3^Institute of Policy and Development Research (IPDR) and College of Education, Hawassa University, Hawassa, Ethiopia

**Keywords:** validity, item development, classroom engagement, EFA, CFA, Ethiopia

## Abstract

**Introduction:**

Classroom engagement is the most influential variable in predicting students’ academic achievement. However, a valid and reliable instrument for measuring students’ classroom engagement in the Ethiopian context remains unexplored. This study aimed to develop and validate an Ethiopian secondary school student classroom engagement instrument.

**Method:**

A total of 119 items were selected through a rigorous literature review, and 40 of these items were initially drafted on the basis of expert judgment. These selected items were subsequently tested on 1,771 students: 383 for exploratory factor analysis (EFA), 1,346 for confirmatory factor analysis (CFA), and 42 for test–retest reliability. The internal consistency of the full scale and subscales of this tool were computed via Cronbach’s alpha. The measurements of invariance across gender and grade levels were analyzed to determine the level of equivalence of the instrument.

**Results:**

The findings revealed that this tool is valid and reliable and measures six sub-constructs of the attribute of classroom engagement. Therefore, the use of such valid and reliable tools in future measurement studies of Ethiopian high school students’ mathematics and science classroom engagement is suggested.

**Conclusion:**

Finally, scholars in math and science research would benefit from using this tool either to cross-validate or synthesize their studies.

## Introduction

Students’ school lives will be more enjoyable and satisfying if they are directly engaged in learning inside and outside their classes. In particular, classroom engagement provides an energetic resource for coping with the challenges of schoolwork, and promote students’ motivational resilience (Martin and Marsh, 2009). In the long term, classroom engagement is a predictor of many important variables in the academic context, such as performance (Archambault and Vandenbossche-Makombo, 2014), academic adjustment (Núñez and León, 2019), psychological wellbeing (Wang et al., 2015), and classroom discipline (Hagenauer et al., 2015).

The question, “what is student engagement?” is surprisingly difficult. In order to address this question various literatures were studied, yet one cannot find a fully agreed upon conceptualization. As a result, different definitions of classroom engagement exist hence; the way how one define it will determine its value for diverse scholars and researchers. Defining it broadly will make it more valuable for the policy making and educated lay thinker groups but less beneficial for the research and academic community. Defining it narrowly will have the opposite effect. Defining it broadly will enhance the overlap of interaction with other ideas and research literatures, making its unique contribution less evident. Defining it narrowly will push “engagement” scholars to make its distinctive contribution and value-added evident (Sinatra et al., 2015).

There is no doubt that “engagement” is currently a very hot topic in the broad field of school achievement. But what is engagement? Researchers yield a range of response to this question: student engagement was defined by Günüç and Kuzu (2014) as “the quality and quantity of students’ psychological, cognitive, emotional and behavioral reactions to the learning process as well as to in-class/out-of-class academic and social activities to achieve successful learning outcomes.” Student engagement has defined as “the time and effort students put on which are related with learning outcomes and academic establishments make sure that students are encouraged to participate designed activities” (Kuh, 2009). Engagement also defined as “students’ psychological investment in and effort directed toward learning, understanding, or mastering knowledge, skills, or crafts that academic work is intended to promote” (Newman et al., 1992). Other studies looked as student engagement more broadly, but broke the construct into sub-components.

According to recent scholarship (e.g., Appleton et al., 2008; Fredricks et al., 2004; Jimerson et al., 2003; Wang et al., 2011), student engagement is a meta-construct that encompasses three dimensions: behavioral, emotional and cognitive. A few studies have defined engagement in terms of its opposite role, disengagement, which is primarily measured and operationalized through disruptions, inactivity, and off-task behaviors (Donovan et al., 2010; Hayden et al., 2011; Rowe et al., 2011). However, a study has suggested that disengagement, or disaffection, is a unipolar notion in and of itself rather than just the bipolar opponent of engagement. Disengagement involves more than just not being engaged; it can also involve actions such as purposefully avoiding tasks, causing disturbances, and expressing unfavorable emotions such as annoyance, indifference, and discomfort (Skinner et al., 2009).

There has been lack of consensus among scholars on definition and components of student classroom engagement (Sinatra et al., 2015). For this study classroom engagement defined as the quality and quantity of students’ cognitive, emotional, and behavioral reactions to the learning processes in class academic activities to achievement successful learning outcomes. In this study classroom engagement categorized into cognitive, emotional, and behavioral components, each component contains both positive and negative reaction of the students.

Behavioral engagement refers to observable behavior which is indicated that students are actively involved to mathematics class, such as time-on task, overt attention, classroom participation, completing class exercise, question asking, expressing idea, and choice of challenging tasks. Behavioral disengagement refers to behavioral disaffection such as behaviors include disruptive classroom behavior, inattention, withdrawal from learning activities, and lack of academic effort (Wang and Degol, 2014).

Emotional engagement is an internal aspect of student engagement and affective reactions to learning activities within the context of classroom environment, such as feeling of interest, enjoyment, happiness, enthusiasm, and perceived value of learning. Emotional disengagement is negative emotions include emotional states of boredom, unhappiness, frustration, and anxiety when involved in classroom learning activities (Whitney and Peterson, 2019).

Cognitive engagement refers to students who invest in their own learning, who accordingly determine their needs and who enjoy the mental difficulties, willingness to expend effort and long period of time to comprehend a subject deeply or master a difficult skill, preservance, investment in learning, value given to learning, learning goals, self-regulation and planning. Cognitive disengagement refers to cognitive disaffection such as actively avoiding work, being disruptive and involving (Fredricks et al., 2004).

In science, technology, engineering, and mathematics (STEM) education, adolescents’ academic progress and choice of college degrees and jobs are significantly influenced by their engagement in math and science classes (Maltese and Tai, 2010; Wang and Degol, 2014). Nevertheless, studies have revealed a decrease in secondary school math and science engagement, particularly among minority and low-income students (Martin et al., 2015). Student engagement is an indication of a motivational state of being in learning rather than a fixed characteristic trait of learning (Sinclair et al., 2003; Skinner et al., 2009). This makes student engagement malleable and open to the influence of interventions conducted within the school environment. To intervene in student engagement, an instrument that appropriately measures student engagement in mathematics and science classes is needed.

There is a scarcity of instruments for measuring student engagement in science and mathematics classrooms. Among the significant number of studies conducted on student engagement measurement tools, a review reported between 1979 and May 2009 of student engagement instruments by Fredrick et al. (2011) identified 156 published instruments. Among those instruments, only one measured student engagement in math and science classes (Kong et al., 2003).

Years later, a few more instruments were designed: the Math and Science Engagement Scales by Wang et al. (2016). This instrument also adapted to Turkish and validated for use in science courses. This scale, consisting of four dimensions (cognitive, behavioral, emotional, and social engagement), showed acceptable validity evidence and strong reliability, making it appropriate for assessing student engagement in math and science classes (Turan Gürbüz et al., 2020). Similarly, Student Engagement in Science Learning (SESL) instrument was developed to measure cognitive, emotional, behavioral, and agentic engagement in the context of China. This instrument demonstrated strong construct validity and reliability, indicating its effectiveness in measuring student engagement in science classroom learning (Li et al., 2024). The Participation and Engagement Scale (PES) was designed to assess student engagement in STEM activities in Italian context. This instrument consists of two factors (satisfaction toward the activities and values of the activities) showing good model fit and reliability through factor and Rasch analysis (Testa et al., 2022).

The following significant gaps were identified in existing student engagement in math and science instruments. Conceptually, all of the tools failed to account for student disengagement in math and science classrooms. Disengagement is currently one of the most major reasons contributing to poor math and science performance. Methodologically, in analyzing the psychometric properties of their instruments, they overlooked test–retest and composite reliability. To obtain the necessary information on the consistency of the instruments, test–retest and composite reliability are critical. Contextually, all of the existing instruments were not specifically designed for high school students; some were designed for middle school students, while others were designed for higher institute students. This study was designed to fill in the gaps in a newly constructed student classroom engagement scale in Ethiopian context.

Research related to student classroom engagement in mathematics and science classes in the Ethiopian context is scarce. Some existing studies that have focused on this issue include the following: upper primary students’ engagement in active learning (Tuji, 2006); creating a context for engagement in mathematics classrooms (Darge, 2006); the affective side of mathematics education; adapting a mathematics attitude measure to the context of Ethiopia (Semela, 2012); the impact of study habits, skills, burnout, academic engagement and responsibility for academic performance (Endawoke and Gidey, 2013); mathematics attitudes among university students; implications for science and engineering education (Zeleke and Semela, 2015); and assessments of the level of student engagement in deep approaches to learning (Tagele, 2018).

One of the major challenges in student classroom engagement research in the Ethiopian context is the lack of uniformity in terms of methodological approaches, especially with respect to the data collection instruments used. While most of these studies used adapted instruments, very few provide adequate descriptions of how the measures were adapted and what procedures were used to contextualize the instruments to local realities. The notable exception in this regard is the research conducted by Semela (2012) titled “the Affective side of mathematics education: adapting a mathematics attitude measure to the context of Ethiopia” in terms of a clear description of the process of adaptation and validation.

The following were the main justifications for developing a new tool for student engagement in the Ethiopian math and sciences classroom context. The primary reason was the absence of a tool capable of simultaneously measuring high school students’ engagement and disengagement. Secondly, there was no validated instrument available that assessed students’ engagement in mathematics and science within an Ethiopian context. The other basic justification for developing a new engagement instrument was the absence of information on the current state of student engagement at both the national and regional levels.

The main purpose of this research is to develop a high school classroom engagement instrument for use in the Ethiopian context, which is suitable for researchers who are interested in this area.

## Theoretical and conceptual framework

Since motivation is seen to be a key component of engagement, studies on classroom engagement have prompted evaluations of the nature of motivation (Klauda and Guthrie, 2015; Reschly and Christenson, 2012; Reeve, 2012; Skinner et al., 2008; Fredricks et al., 2004; Bomia et al., 1997; Finn, 1989; Voelkl, 1997). Motivation and engagement are inextricably intertwined; according to the 2004 National Research Council and Institute of Medicine report Engaging Schools. Therefore, it is believed that a person’s degree of engagement is directly influenced by their motivational quality, with higher levels of engagement resulting from more intrinsic motivation (Connell and Wellborn, 1991; Ryan and Deci, 2000; Siu et al., 2014).

There are several theories of motivation which give much emphasis for engagement and disaffection in their discussion of motivational process. For this research Self-determination theory was selected as theoretical framework to conceptualize student engagement.

**Figure 1:**
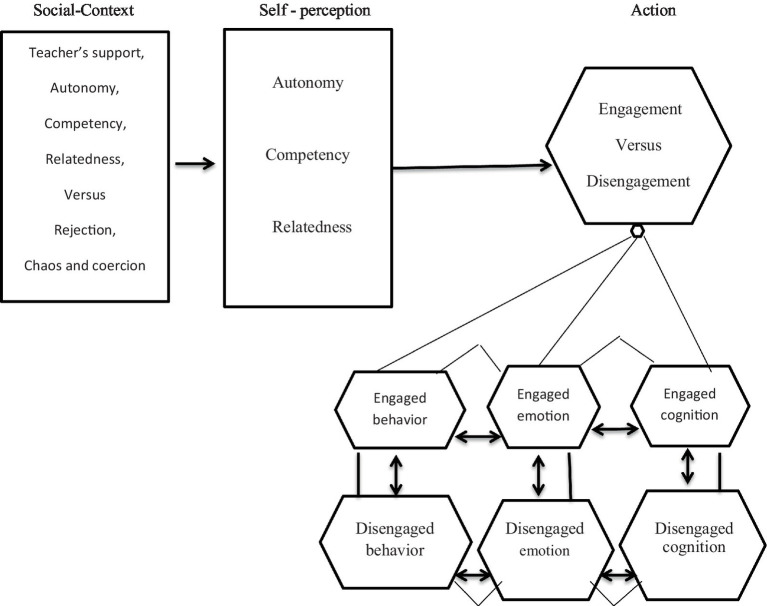
Conceptual model.

Self-determination theory (SDT), proposed by Deci and Ryan (1985), which holds that all people have three basic, universal psychological needs: relatedness (a sense of being loved and connected to others), competence (a sense of being effective and competent), and autonomy (a sense of being self-governed and self-initiating in activities). People experience greater psychological wellbeing when these three demands are met; when they are not, they become more reactive, isolated, and severely fragmented. People are more likely to be engaged in pertinent activity when their psychological needs are satisfied by interactions with others in their social setting. Classrooms that support these three psychological needs are more likely to engage students in learning tasks (Reeve, 2013). SDT offers a theoretical framework for understanding how learners’ motivational experiences might be impacted by the social setting of the classroom (Ryan and Deci, 2017). Nevertheless, the role that classroom engagement plays in the learners’ motivational system is not well explained by this theory. The Self-System Model of Motivational Development (SSMMD) was proposed as a conceptual framework by the researchers (Skinner et al., 2008; Skinner et al., 2009) in order to establish a causal relationship between classroom engagement and other motivational variables identified by SDT. This model includes four categories of motivational variables. The social environment of students is the first component, which includes peers, parents, and teachers, is referred to as a context variable. Learners’ self-perception is the second component which includes abilities beliefs, values, and attitudes—and in particular, how well their needs for autonomy, competence, and relatedness are met—are referred to as self-variables. Action, the third component, relates to goal-directed actions, including participation in educational activities. The model’s final element is the outcome, which in the context of education is represented by learning and cognitive growth. With its four components, the SSMMD explains how the setting influences the fundamental psychological requirements that SDT identified as significant facets of the self, which in turn influences engagement and pertinent results. We used this comprehensive model of the motivational process, which is illustrated in Figure 1, for current study.

## Methodology

This study aimed primarily to develop and validate a student classroom engagement instrument for high school students in the Ethiopian context. Therefore, this study was conducted in two phases: instrument development and instrument validation.

### Study procedure

**Figure 2:**
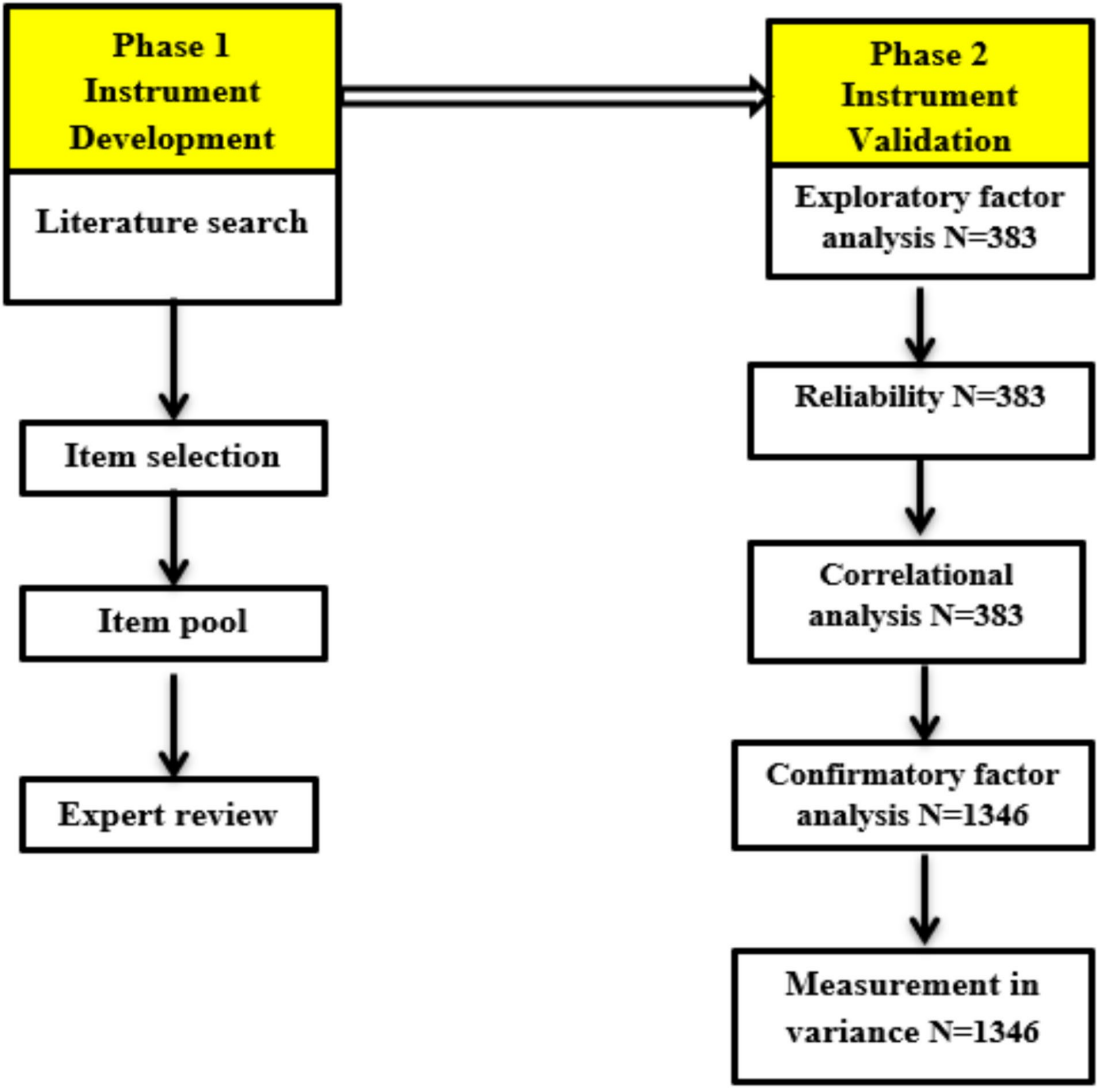
Instrument development processes.

This instrument was developed and validated on the basis of DeVellis and Thorpe (2021) scale development guidelines. The first phase begins with a literature review with the aim of defining student classroom engagement and identifying dimensions and measurements of student classroom engagement instruments. After an initial item pool was created, a group of specialists was gathered to discuss the relevance, clarity, and cultural appropriateness of each item; each step is described below. During the second phase (instrument validation), the instrument’s psychometric qualities were evaluated via exploratory factor analysis, internal consistency, confirmatory factor analysis, and tests of measurement invariance; Figure 2 illustrates the process. The psychometric qualities were investigated with two different participant groups.

### Item selection

To obtain items for the newly developed scale, a significant number of studies from different sources were collected and reviewed. Only relevant numbers of articles, documents and reports that contain measurements and definitions of student classroom engagement in science and mathematics were selected for use in this study. A review of the selected literature revealed the following: ‘Math and Science Engagement Scale-MSES’ (Wang et al., 2016); ‘Student Engagement Scale- SES’ (Günüç and Kuzu, 2014); ‘Scale of Student Engagement in Statistics-SSES’ (Whitney et al., 2019); ‘Motivational and Engagement Survey’ (Liem and Martin, 2012); ‘Engagement versus Disaffection with Learning-EvsD-scale’ (Skinner et al., 2008); ‘Survey Items Related to Student Engagement- SIRSE’ (Fredricks and McColskey, 2012); ‘Student Engagement in Mathematics Classroom-SEMC’ (Kong et al., 2003); and ‘Student Engagement Instrument-SEI’ (Appleton et al., 2006). Self-report tools were used as major sources to select items for this newly developed Ethiopian student engagement instrument.

### Item pool

From the aforementioned eight instruments, a total of 141 items were selected on the basis of the consensus of the corresponding author and the third coauthor, who were again cross-checked and affirmed by the second coauthor of the manuscript for use as an initial pool of items for the newly developed instrument. The pool of items was subsequently classified into cognitive, emotional, and behavioral dimensions. Here, the items’ factorial positions in the original instruments are given due emphasis during classification or labeling. After these items were categorized under each factor, redundant items were removed. One of the greatest challenges encountered during the categorization of items was the proven existence of the same item in different components or factors in the eight selected tools. To avoid such situations, the conceptualization and operationalization of the attribute, engagement in general, and its sub-constructs in particular were carefully referred to and analyzed concomitantly with simple inspection of the number of times a particular item suited in one or more engagement factors in the aforementioned tools that have been used as the basis of categorization.

In addition, item clarity, appropriateness, validity, reliability and cultural appropriateness were used as additional selection criteria. After passing the required selection procedures, 119 items were believed to be appropriate by the authors of the manuscript for measuring student classroom engagement in science and mathematics in the context of Ethiopia. These items were selected and made ready for carefully chosen experts’ ratings of their relevancy, clarity, and cultural appropriateness, as discussed below.

### Expert review

Five experts were selected on the basis of their experience and educational background. Two of the experts were assistant professors in educational counseling. They conducted research on study habits, factors affecting classroom achievement and the impact of motivation on classroom achievement. The other two experts were PhD mathematics candidates with more than 10 years of teaching experience in high schools. The remaining expert was a master’s degree in measurement and evaluation with ample experience in teaching mathematics. First, definitions, conceptualizations, measurement procedures, and personality item writing principles were discussed with these experts, and selected reading materials were also shared.

Following the above procedures, these experts rated the relevance, clarity and cultural appropriateness of each item, and they were given complete freedom to modify, correct and recommend improvements to each item.

With respect to item clarity, the experts tried to evaluate each item in terms of how easily the item was understood by students and how free it was from jargon and ambiguity. Once again, these experts rated each item’s relevance and cultural appropriateness on a similar number of response options as they did for item clarity. For all three parameters, clarity, relevance, and cultural appropriateness, the ratings had two options: “YES” for those items they agreed with and “NO” for those they disagreed with.

Items that did not receive acceptance by even one of the experts in any of the criteria were removed. In addition, on the basis of the information obtained from the experts, items in different categories were corrected, restated or deleted. The final set of items was subsequently approved for language translation.

### Item translation

After the necessary corrections were made to the final selected items, translation with contextualization from the original English version into Amharic was performed by two language experts; both are assistant professors of the Amharic language and literature. The translation and contextualization were performed independently, and discrepancies were resolved through consensus. In addition, to understand how this instrument is a valid tool for measuring students’ engagement, two carefully selected instruments measuring similar variables as the new tool were translated, pilot tested and made ready for final administration, which aimed to compute the concurrent validity. The first instrument consists of 15 items from the ‘Korean Basic Psychological Needs Scale-KBPNS’ (Lee and Kim, 2008; Kim et al., 2021), and the second consists of 24 items from the ‘Teacher as Social Context Questionnaire-Student Report-*TASCQ-S*’ (Skinner and Belmont, 1993). The full scale of KBNS has three factors, autonomy, competence, and relatedness, with five items under each factor. However, the full scale of the TASCQ-S again has three sub-constructs, autonomy *s*upport, structure, and involvement, with eight items under each factor.

### Data analyses techniques

Experts’ judgments were used to obtain evidence about the content validity of the instrument and the internal validity of this tool. EFA, CFA, and Pearson product moment correlation were computed to ensure internal validity. In addition, the Pearson product moment correlation coefficient was also used to obtain evidence of the validity of how the student classroom engagement instrument correlates with theoretically related variables. Furthermore, multigroup CFA was computed to determine whether the student classroom engagement measurement was invariant in terms of gender and grade level. Cronbach’s alpha and test–retest and inter item correlations were used to determine the internal consistency and reliability of the instrument. The statistical analyses of this study were computed via SPSS version 20 and SPSS with Amos version 23.

### Participants

The data collected from two secondary schools, Dilla secondary school and Community School, found in Dilla Town, southern Ethiopia, were used to perform exploratory factor analysis (EFA), Cronbach’s alpha, and relationships among the variables. The number of students in Community secondary schools was very small, i.e., only 263; thus, all the students were considered in this study. At Dilla Secondary School, however, the number of students was more than 2,000. Using simple random sampling, 208 students who were available at the time of data collection from four randomly selected sections—one from each 9th, 10th, 11th and 12th grade—were included in this study. The final data were collected from all 383 students from the above government and community secondary schools found in Dilla Town.

Confirmatory factor analysis (CFA) and multi-group confirmatory factor analysis (MGCFA), data were computed on a random sample of 1,620 students from two governments and two private secondary schools in Dilla town and Hawassa city. The numbers of students in both private schools were very small, so all the students were included in this study. However, the numbers of students in both government schools were very high; thus, a simple random sampling method was employed to select two sections from each secondary grade: 9th, 10th, 11th and 12th. Finally, after the data were collected, some participants who left either a significant number of items or the entire set blank and who failed to specify their sex, grade, or school type were excluded from the final analysis of this study. Accordingly, data were collected from 1,348 students and used for CFA and MGCFA statistical analysis. Two students who did not respond to all of the items were also excluded. The analyses of this study were conducted on the basis of the responses of 1,346 students.

With respect to test–retest reliability, data were collected from 42 grade 10 students (24 females and 18 males) at the Hambiwol secondary school in Dilla town via a simple random sampling technique (lottery method). Tables 1, 2 present the information of the participants and their distributions for the EFA, CFA, and MGCFA.

**Table 1:** Distribution of the participants for the EFA.

Grade	School type				
	Community Secondary School		Dilla Secondary School		Total
	Gender		Gender		
	Male	Female	Male	Female	
9th	34	40	20	26	120
10th	25	31	18	14	88
11th	20	41	23	16	100
12th	14	12	27	22	75
Total	93	124	88	78	383

**Table 2:** Distribution of the participants for the CFA and MGCFA.

Grade	School type				
	Government		Privet		Total
	Gender		Gender		
	Male	Female	Male	Female	
9th	71	85	102	108	366
10th	81	110	76	106	373
11th	138	72	74	82	366
12th	73	36	65	67	241
Total	363	303	317	363	1,346

On the basis of Tables 1, 2, the data collected from these sample of students demonstrated a balanced gender representation: 680 females (50.5%) and 666 males (49.5%). Similarly, the percentage of students from different school types was balanced: 666 of the students were from government schools (49.5%), and 680 were from private schools (50.5%). Additionally, the students who participated in this study came from different grade levels: 9 (*n* = 366, 27.2%), 10 (*n* = 373, 27.7%), 11 (*n* = 366, 27.2%), and 12 (*n* = 241, 17.9%). With respect to the student ratios of gender groups, school types and grade levels, the sample could be considered not biased.

## Results

This section presents the results of, exploratory factor analysis (EFA), confirmatory factory analysis (CFA), multi-group CFA and zero-order correlations. In addition, the assessment of internal consistency reliabilities (Cronbach’s alpha) of the adapted instruments will also be presented and discussed.

### Exploratory factor analysis

One of the basic tasks in the instrument development process is identifying the most parsimonious factor structure. Factor analysis highly depends on the number of participants. Nunnally and Bernstein (1994) recommend a sample size of at least 300 respondents to apply factor analysis effectively. In this study the number of respondents exceeds the recommended sample size (*n* = 383) which was adequate to use factor analysis effectively.

Furthermore, factor structures were evaluated in line with the widely applied criteria (see: Nunnally and Bernstein, 1994; DeVellis and Thorpe, 2021). These criteria are as follows: (a) to determine sample size adequacy, the value of Kaiser–Meyer–Olkin (KMO) measure for sample adequacy should be greater than 0.60 and Bartlett’s Sphericity (multivariate normal distribution indicator) should be close to 0; (b) item factor loadings should be greater than or equal to 0.4; (c) the eigenvalues of the un-rotated factors should be greater than or equal to one; (d) the number of items in one factor should be at least three; and (e) the degree of the variance accounted for by a factor in relation to the total scale variance should be 50 percent or greater.

The data were suitable for exploratory factor analysis, with the Kaiser–Meyer–Olkin measure for sample adequacy being.992 and Bartlett’s test of sphericity = 5,213.920, with df = 780 and *p* < 0.0001.

**Figure 3:**
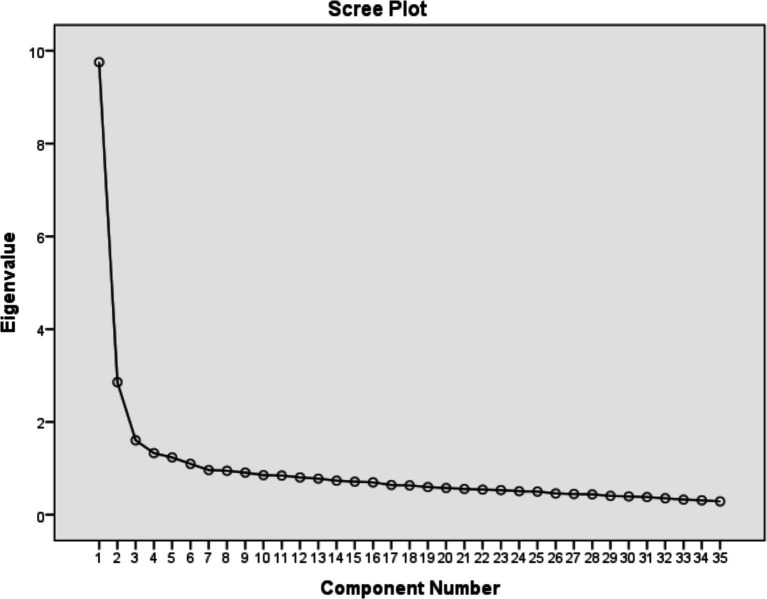
Scree plot.

A principal component analysis (PCA) was conducted on the 40 items with orthogonal rotation (varimax). This analysis resulted in six components made up of 35 items each with eigenvalues were greater than 1. In addition, the PCA yielded components each made up of three or more items. However, the factor analysis eliminated five items (1, 2, 3, 8, and 16) due to low factor loadings. The screen plot (Figure 3) shows inflexions that would justify the retention of six factors. Table 3 shows that the six factors combined together explain 51.068% of the total variance; the results are presented below.

**Table 3:** Total variance explained for EFA data.

Component	Initial eigenvalues			Extraction sums of squared loadings			Rotation sums of squared loadings		
	Total	% of Variance	Cumulative %	Total	% of Variance	Cumulative %	Total	% of Variance	Cumulative %
1	9.754	27.868	27.868	9.754	27.868	27.868	5.390	15.401	15.401
2	2.858	8.165	36.033	2.858	8.165	36.033	3.422	9.777	25.178
3	1.603	4.579	40.612	1.603	4.579	40.612	2.986	8.531	33.708
4	1.327	3.792	44.404	1.327	3.792	44.404	2.584	7.382	41.090
5	1.235	3.529	47.933	1.235	3.529	47.933	1.921	5.488	46.578
6	1.097	3.135	51.068	1.097	3.135	51.068	1.571	4.490	51.068

Extraction Method: Principal Component Analysis.

KMO Sampling Adequacy = 0.992.

Bartlett Test of Sphericity = 5,213.920, *p* < 0.0001; df = 780.

Table 4 demonstrated the rotated component matrix indicates how the items are distributed in different components.

**Table 4:** Rotated component matrix.

No	Items	Component					
		1	2	3	4	5	6
36	If given, I identify key information from any reading assignment on science and math lessons	0.705					
34	I combine ideas from different courses to help complete my science and math assignments.	0.678					
38	I look for chances to be part of science events that are related to things we are doing in my science class.	0.657					
39	I look for extra information (books or internet) to learn more about things we do in science classes.	0.633					
37	When I learn a new science lesson, I ask myself questions to make sure I understand what I am learning about.	0.629					
35	I summarize the material I learn in class or from other course materials.	0.626					
32	I spend enough time and make enough effort to learn science and math	0.624					
33	When I am studying science and math lessons, I try to connect different topics from course material.	0.621					
31	I prepare for science and math courses before going class.	0.619					
40	If I do not understand what I read in science and math classes, I go back and read it over again, look it up, discuss it with someone.	0.486					
28	I look different ways to solve science and math problems.	0.433					
6	I keep trying even if something is hard in science and math courses		0.651				
9	I put effort into learning science/math individually and group		0.643				
11	I try to do my best regarding my responsibilities in group work on science and math courses		0.632				
12	In science and math class, I work as hard as I can.		0.6				
14	When I am in science and math classes, I listen very carefully.		0.572	.			
15	I enjoy learning new things about science and math.		0.557				
7	I make sure to study on a regular basis science and math courses		0.511				
10	I take good notes in class, on readings, and/or on video lectures.		0.419				
21	I am motivated by my desire to learn math and science.			0.63			
20	I am very interested in learning science and mathematics.			0.617			
23	My science and math classwork makes me curious to learn other things.			0.588			
25	I feel excited by the learning activities in my science and math classes.			0.587			
26	When I am in science and math classes, I feel good.			0.572			
22	I find ways to make science and math course interesting to me.			0.551			
18	I do not care about learning science and math.				0.748		
17	I do not want to be in science and math classes.				0.697		
19	I often feel down when I am in science and math classes.				0.641		
24	I feel bored when I am learning science and math.				0.528		
30	When science and math work is hard I only study the easy parts					0.714	
29	During science and math classes, I would rather be told the answer than have to master the procedure.					0.628	
27	When I am working on my science and math classwork, I feel disgusting.					0.557	
5	I do not try to work very hard at science and math courses						0.71
4	I do irrelevant things when I am supposed to be paying attention in science and math classes						0.605
13	When I am in science and math class, I just act as if I am working.						0.474

On the basis of the nature of the items loaded on the same component and the literature review, the name of each component was given. Table 5 shows the items with the corresponding component names.

**Table 5:** Components of student classroom engagement.

Component	Items	Name of the component
Component 1	36, 34, 38, 39, 37, 35, 32, 33, 31, 40 and 28	Cognitive engagement
Component 2	6, 9, 11, 12, 14, 15, 7 and 10	Behavioral engagement
Component 3	21, 20, 23, 25, 26 and 22	Emotional engagement
Component 4	18, 17, 19 and 24	Emotional disengagement
Component 5	30, 29 and 27	Cognitive disengagement
Component 6	13, 5 and 4	Behavioral disengagement

### Confirmatory factor analysis

After conducting the EFA, which determines the factor pattern of the student classroom engagement instrument, it is desirable to perform the CFA of the model as well. CFA is used to collect evidence about whether a hypothesized factor model does or does not fit the dataset. CFA is the most powerful analysis used to assess whether a predefined factor model fits the data (Floyd and Widaman, 1995; Netemeyer et al., 2004).

The structure of the student classroom engagement instrument, which consists of 35 items and six factors, was tested via confirmatory factor analysis. This analysis was performed with 1,348 students who were selected randomly. Factor loading for each item was evaluated as part of the confirmatory factor analysis process. Because of their low factor loading (<0.5), items 6, 18, and 33 were eliminated. The items for CFA were presented in Annex 2. Furthermore, the Amharic version of the items were presented in Annex 3.

**Figure 4:**
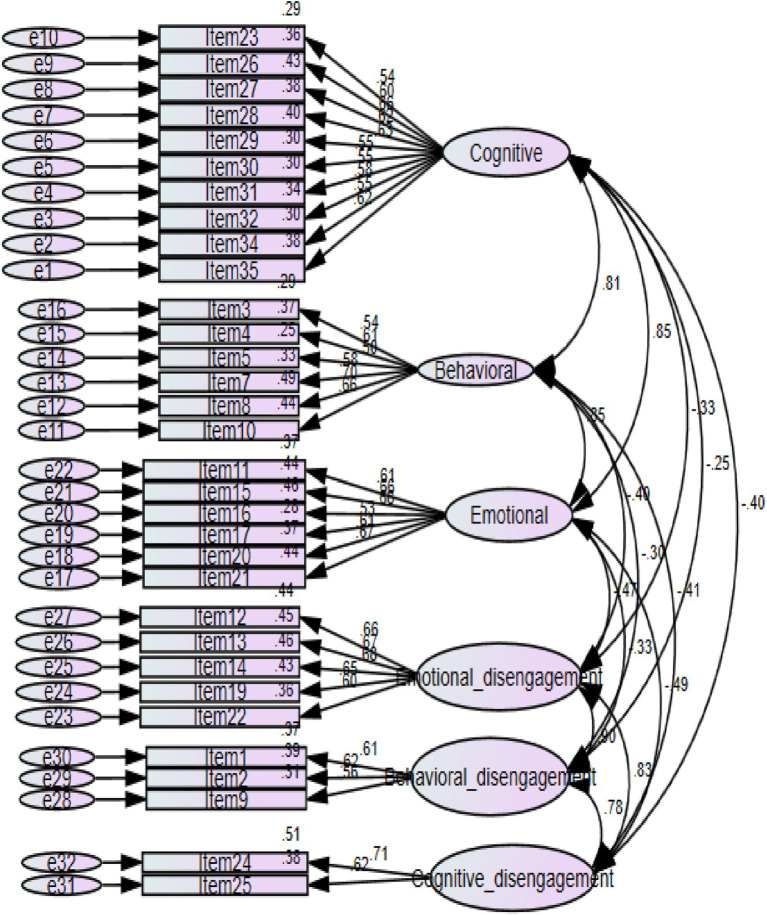
CFA six-factor model structure.

Figure 4 indicated the six-factor model (cognitive engagement, behavioral engagement, emotional engagement, cognitive disengagement, behavioral disengagement, and emotional disengagement) yielded good fits for the data: CMIN/df = 3.14, GFI = 0.939, CFI = 0.928, TLI = 0.922, SRMR = 0.0369, RMSEA = 0.040.

### Measurement invariance

**Figure 5:**
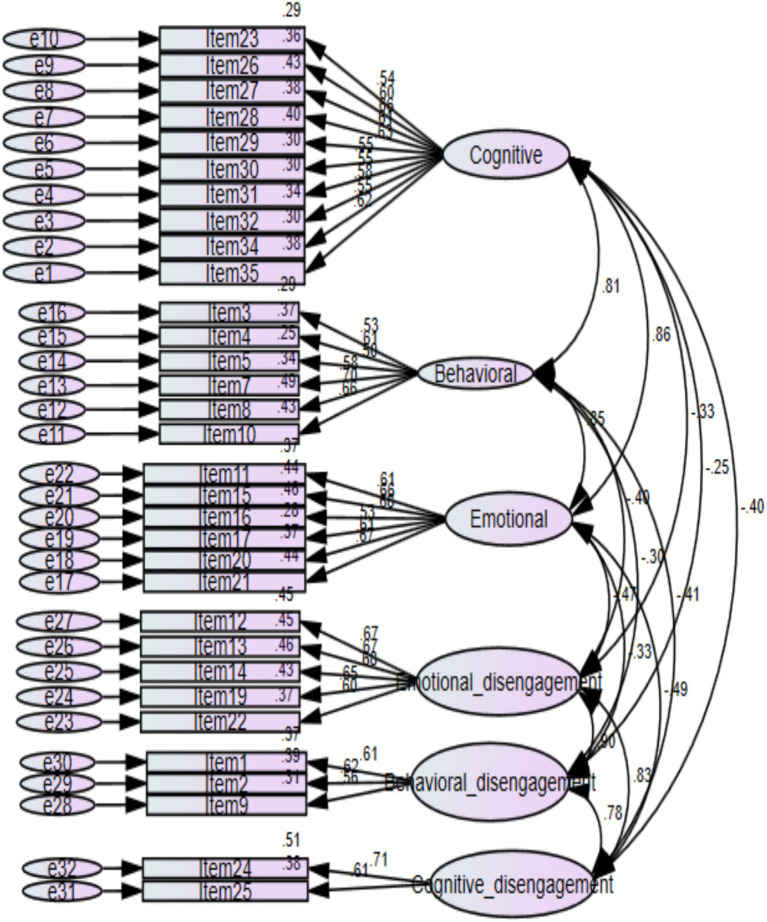
MGCFA six-factor model structure for gender.

Measurement invariance is a property of an instrument and confirms that an instrument does indeed measure the same construct in the same way across different groups (Schmitt and Kuljanin, 2008). Multigroup confirmatory factor analysis (MGCFA) was used to determine whether the student classroom engagement measure was invariant in terms of gender and grade level. When the MGCFA is applied, a series of three hierarchically ordered steps are addressed. The first step is configural invariance; this step is used as a baseline for fit comparison for later steps of measurement invariance, and no invariance constraints are imposed. Second, metric invariance is tested by constraining factor loading (i.e., the loading of items on the constructs) to be equivalent across gender and grade levels. Finally, the scale step is addressed by factor loading; here, intercepts are constrained to be invariant across gender and grade levels (Figure 5).

To decide on measurement invariance across gender and grade level, comparisons were made between the constrained and unconstrained models in a stepwise process. Since the configural invariance is an unconstrained model, it is tested by evaluating the overall fitness of the model. To test the configural model, the following fit indices were used: CFI >0.9, (Bentler, 1990) RMSEA and SRMR <0.08, (Hu and Bentler, 1998). Accordingly, comparisons were made between the configural invariance model and the metric invariance model and, finally, between the metric invariance model and the scalar invariance model. To test comparisons among the models, (Chen, 2007) cutoff points for measurement invariance were used (ΔCFI ≤ 0.01, ΔRMSEA and ΔSRMR ≤ 0.030).

### Invariance with respect to gender

To determine the measurement equivalence of the instrument with respect to gender, 666 female and 680 male high school students were used. Multigroup comparisons among the models were used to assess invariance. The table below presents a detailed multigroup comparison of the fit indices.

Table 6 shows the configural invariance of the student classroom engagement instrument at an acceptable level (CFI > 0.9, RMSEA and SRMR < 0.08). This finding indicated that the factor structure of the instrument was similar for male and female students. To determine the metric invariance of the instrument, comparisons were made between the configural and metric models. The change statistics of the comparison indicated that the metric invariance was supported (ΔCFI < 0.001, ΔRMSEA < 0.001, ΔSRMR < 0.001). This showed that not only the factor structure but also the factor loading of the items was equivalent for male and female students. To determine scalar invariance, a comparison was made between the metric and scalar models. The change statistics confirmed that scalar invariance was supported (ΔCFI < 0.003, ΔRMSEA < 0.001, ΔSRMR < 0.001). This result implied that in addition to factor loadings, the item intercepts are equivalent for male and female students. In general, the instrument is invariant in all three stages of measurement invariance. In other words, the results confirmed that the student classroom engagement instrument has measurement equivalence with respect to gender.

**Table 6:** Measurement invariance analysis of the instrument for gender groups.

Level of invariance	DF	CMIN	CMIN/DF	*p*	CFI	RMSEA	SRMR	ΔCFI	ΔRMSEA	ΔSRMR
Configural	898	1,915.761	2.133	0.000	0.926	0.029	0.0415			
Metric	924	1,944.413	2.104	0.000	0.926	0.029	0.0425	0.000	0.000	0.001
Scalar	956	2,007.822	2.100	0.000	0.923	0.029	0.0425	0.003	0.000	0.000

p = Associated probability; CMIN = Minimum Discrepancy Function; df = degree of freedom; CFI = Comparative Fit Index; SRMR = Standardized Root Mean Square Residual; RMSEA = Root Mean Square Error Approximation; ΔCFI = change in values of CFI; ΔRMSEA = change in values of RMSEA; ΔSRMR = change in values of SRMR.

### Invariance across grade levels

To assess the measurement equivalence of the instrument across grade levels, grade 9 (*n* = 366, 27.2%), grade 10 (*n* = 373, 27.7%), grade 11 (*n* = 366, 27.2%), and grade 12 (*n* = 241, 17.9%) students were used. Multigroup comparisons among the models were used to assess invariance. The table below presents a detailed multigroup comparison of the fit indices.

Table 7 shows that the configural invariance result across grade levels was within an acceptable range (CFI > 0.9, RMSEA and SRMR < 0.08). This revealed that the factor structure of the instrument was similar for students across grades 9–12. The change statistics between the configural and metric steps indicated that metric invariance was supported (ΔCFI < 0.004, ΔRMSEA < 0.001, ΔSRMR < 0.006). This showed that not only the factor structure but also the factor loading of the items was equivalent for students across grades 9–12. In addition, the change statistics between the metric and scalar steps indicated that scalar invariance was supported (ΔCFI < 0.009, ΔRMSEA < 0.001, and ΔSRMR < 0.004). This result revealed that in addition to factor loading, the item intercepts are equivalent for students across grades 9–12. The above results obtained from the three measurement invariance steps prove that the student classroom engagement instrument has measurement equivalence across grade levels.

**Table 7:** Measurement invariance analysis of the instrument for grade levels.

Level of invariance	DF	CMIN	CMIN/DF	*p*	CFI	RMSEA	SRMR	ΔCFI	ΔRMSEA	ΔSRMR
Configural	1,796	3,106.914	1.730	0.000	0.904	0.023	0.0528			
Metric	1,874	3,234.347	1.726	0.000	0.900	0.023	0.0583	0.004	0.000	0.0055
Scalar	1,970	3,453.449	1.753	0.000	0.891	0.024	0.0587	0.009	0.001	0.0004

p = Associated probability; CMIN = Minimum Discrepancy Function; df = degree of freedom; CFI = Comparative Fit Index; SRMR = Standardized Root Mean Square Residual; RMSEA = Root Mean Square Error Approximation; ΔCFI = change in values of CFI; ΔRMSEA = change in values of RMSEA; ΔSRMR = change in values of SRMR.

### Reliability

The consistency of the test scores is evaluated in terms of the reliability coefficient. Three broad categories of reliability coefficients are recognized: alternative-form coefficients, test–retest coefficients, and internal-consistency coefficients.

There are inconsistencies among scholars regarding the appropriate value to justify reliability results. Zeller (2005) suggested that a Cronbach’s alpha of 0.9 or higher is considered excellent; 0.80–0.90 is adequate; 0.70–0.80 is marginal; 0.6–0.70 is seriously suspicious; and less than 0.6 is unacceptable. While (DeVellis and Thorpe, 2021) indicated that results less than 0.6 are unacceptable, those between 0.60 and 0.65 are undesirable, those between 0.65 and 0.70 are minimally acceptable, those between 0.70 and 0.80 are respectable, those between 0.80 and 0.90 are very good, and those much above 0.9 should be considered to shorten the scale. In contrast, Nunnally and Bernstein (1994) reported that a satisfactory level of reliability depends on how a measure is being used. In the early stage of validation research, only a modest reliability of 0.70 is sufficient.

### Internal-consistency reliability

To assess the reliability of the student classroom engagement instrument’s internal consistency, a procedure was used, and the results are presented below.

Table 8 shows that internal consistency reliabilities (Cronbach alpha values) for all components and for the full-scale ranged between 0.660 and 0.904 and the composite reliability were greater than 0.80, exceeding the threshold limit of 0.70 (Hair et al., 2019), thereby the newly developed student classroom engagement instrument has very good internal consistency.

**Table 8:** Cronbach alpha and composite reliability.

Constructs	Number of items	Cronbach’s Alpha	Composite reliability
Behavioral engagement	10	0.818	0.830
Cognitive engagement	6	0.881	0.860
Emotional engagement	6	0.797	0.850
Behavioral disengagement	5	0.649	0.847
Cognitive disengagement	3	0.755	0.860
Emotional disengagement	2	0.660	0.900
Full scale	32	0.904	

### Test–retest reliability

To assess the stability of student classroom engagement scale, test–retest reliability was assessed twice. Thirty-five items were selected after EFA. The 35 items were given to 42 Grade 10 students who were selected from two secondary schools. The time interval for the two tests was 7 days (June 8 and June 15, 2023). The test–retest reliability was calculated via Pearson product–moment correlation. It was found to be [*r* (42) = 0.789, *ρ* < 0.001].

### Item–total correlation

Item–total correlations were performed to determine the relationship of the student classroom engagement instrument with individual items. The result (see Annex 1) shows a statistically significant correlation with a range of *r* (383) = 0.224–0.679, *ρ* < 0.01. Except for items 5, 13, 18, and 30, all the other item–total correlations were greater than 0.3. In addition, all the items were significantly correlated with their subcomponent total [383) = 0.542–0.771, *ρ* < 0.01]. Almost all the items’ correlations with their subcomponent totals were 0.6 and above.

### Correlations among the dimension of student classroom engagement

Understanding the relationships among the dimensions of an instrument is another important way to obtain information about the internal structure of the instrument. In this research, correlations among dimensions were also analyzed via the Pearson correlation coefficient, and the following results were obtained.

Table 9 shows how the subscales are related to each other and to the full scale. All the subscales were strongly and significantly related to the full scale in the range of *r* (1383) = 0.476–0.847, *ρ* < 0.01. This implies how those dimensions measure the same construct. The above table also indicates that cognitive, behavioral, and emotional engagements have better relationships with each other [*r* (1383) = 0.633–0.697, *ρ* < 0.01]. Similarly, emotional, behavioral, and cognitive disengagement produced better relationships with each other [*r* (383) = 0.474–0.599, *ρ* < 0.01]. The above table demonstrated that the relationship among engagement and disengagement components were weak and negative [*r* (1383) = −0.137–−0.343, *ρ* < 0.01].

**Table 9:** Subcomponent score correlations.

Components	CE	BE	EE	ED	BD	CD	FS
Cognitive engagement (CE)	__						
Behavioral engagement (BE)	0.646**	__					
Emotional engagement (EE)	0.697**	0.633**	___				
Emotional disengagement	−0.134**	−0.166**	−0.184**	___			
Behavioral disengagement	−0.168**	−0.186**	−0.237**	0.599**	__		
Cognitive disengagement	−0.286**	−0.253**	−0.343**	0.513**	0.474**	__	
Full Scale (FS)	0.847**	0.787**	0.843**	0.616**	0.476**	0.548**	__

### Evidence on relationships to other variables

To determine how this newly developed instrument appropriately measures student classroom engagement, evidence of its relationship with theoretically related variables is needed. To obtain this evidence, teacher support and student need satisfaction were selected on the basis of the theoretical framework in which this instrument was developed. To evaluate how those variables were related to each other, the Pearson correlation coefficient was used.

Student classroom engagement is significantly and strongly related to student need satisfaction [*r* (383) = 0.571, *ρ* < 0.01] and teacher support [*r* (383) = 0.439, *ρ* < 0.01]. This explains why the newly developed student classroom engagement instrument is valid for measuring student classroom engagement in science and mathematics classrooms.

### Convergent and divergent validity

To confirm convergent validity, the average variance extracted (AVE) is assessed, the value at 0.5 or higher is acceptable. As seen in Table 10, AVE values ranged from 0.470 to 0.750, except one construct (Cognitive Engagement) and every other construct were greater than 0.5 (Hair et al., 2019), presenting convergent validity. To assess the divergent validity, Fornell–Larcker Criterion (Fornell and Larcker, 1981) was applied, the square root of the AVE of each latent construct was equated with its inter-construct correlation. Acceptable divergent validity is achieved when the square root of the AVE of a construct is greater than its correlation with other constructs (Hair et al., 2019). As presented in Table 10, divergent validity was supported as the square root of AVE for the construct was more significant than the correlation with other constructs. This indicated that the divergent validity is present.

**Table 10:** Convergent and divergent validity (Fornell-Larcker criterion).

	Number of items	AVE	1	2	3	4	5	6
1. Behavioral engagement	10	0.501	0.708					
2. Cognitive engagement	6	0.470	0.638	0.694				
3. Emotional engagement	6	0.531	0.684	0.686	0.729			
4. Behavioral disengagement	3	0.649	0.234	0.253	0.344	0.806		
5. Cognitive disengagement	2	0.755	0.372	0.441	0.405	0.504	0.869	
6. Emotional disengagement	5	0.644	0.305	0.307	0.472	0.740	0.631	0.802

## Discussion

This section aims to interpret the findings in relation to the research questions identified earlier.

The purpose of this study was to develop and validate a student classroom engagement instrument for Ethiopian high school students. In the process of developing this tool, first, relevant existing tools are reviewed to select items and create a suitable item pool for each component. After this, some important items under each component were modified to make them appropriate in the context of science and mathematics. Additionally, items that were repeated, unrelated, ambiguous, or unclear were discarded from the initial pool. Next, these selected items were given to five experts to evaluate their relevancy, clarity, and cultural appropriateness. On the basis of the experts’ ratings, 40 items were selected for subsequent psychometric evaluation.

### Dimensionality of student classroom engagement

The results obtained from EFA indicated that the newly developed instrument has six valid components. This finding supports student classroom engagement as a multidimensional construct (Appleton et al., 2008; Fredricks et al., 2004; Jimerson et al., 2003; Wang et al., 2011). A multidimensional perspective on student engagement makes it possible to look at the individual effects of each dimension of engagement on math and science outcomes.

Most of the components that are identified in this study, such as cognitive, affective, and behavioral, are observed in the previously designed student classroom engagement instruments: the Scale of Student Engagement in Statistics (SSE–S) (Whitney et al., 2019), the Student Engagement in Mathematics Classroom measure (Kong et al., 2003), and the Student Engagement Scale (SES) (Günüç and Kuzu, 2014). In addition, behavioral and emotional disengagement, as a measure of student classroom engagement, were included in the Engagement versus Disaffection with Learning (EvsD) scale (Skinner et al., 2009). In most previous student classroom engagement scales, disengagement was not measured separately. Moreover, this instrument specifically includes cognitive disengagement as a component of student classroom engagement. It is important to note that disengagement is not only the opposite of engagement, as it refers to different action and it has a multidimensional nature. Disengagement encompassing behavioral, emotional, and cognitive dimensions. This is supported by Self-determination theory (SDT), proposed by Deci and Ryan (1985) and The Self-System Model of Motivational Development (SSMMD) (Skinner et al., 2008; Skinner et al., 2009).

### Psychometric qualities of the instrument

To use the newly developed student classroom engagement instrument effectively, its psychometric quality should be determined. This could be done through establishing validity and reliability evidence.

### Validity

The process of validation involves the accumulation of relevant evidence to provide a sound scientific basis for the proposed score interpretations. To validate the student classroom engagement instrument, validity evidence was obtained for test content, internal structure, and relationships with other variables (Appleton et al., 2006).

**Evidence from the internal structure of the instrument**: is also known as construct validity. The evidence concerning the internal structure of the student classroom engagement instrument was obtained from confirmatory factor analysis (CFA), item–total correlation, and correlation among the components that were extracted.

CFA was performed to confirm the factor structure of the student classroom engagement scale. All indices of the model have acceptable fit values, so it is possible to claim that the structural validity of the student classroom engagement scale is confirmed. In addition, the components that were identified by EFA were valid for measuring student classroom engagement in mathematics and science classrooms. Similarly, Günüç and Kuzu (2014), Skinner et al. (2009), Liem and Martin (2012), and Appleton et al. (2006) used CFA as a criterion to determine the validity of the instrument that was designed.

Evidence about the internal structure of an instrument can be obtained by determining the extent to which items tend to measure the same construct. To understand how the items in this instrument measure the same thing, item–total correlations for the whole scale and subscales were used. All the items were statistically significant across the whole scale and subscale at *α* = 0.01, with correlation values ranging from [*r* (383) = 0.224–0.771; *ρ* < 0.01]. The above results indicate that all the items measure the same construct across the whole scale and the subscales.

How the components interrelate with each other and the whole scale indicate the internal structure of the instrument. The newly developed student classroom engagement instrument has four components, each of which has a statistically significant correlation with the whole scale at *α* = 0.01, with correlation values ranging from [*r* (1348) = 0.32–0.865; *ρ* < 0.01] (Colton and Covert, 2007) and (DeVellis and Thorpe, 2021) acknowledged that stronger correlations among the components and the whole scale were sources of evidence for the internal structure of the instrument.

**There is evidence of a relationship with other variables:** before we use the newly developed instrument, there needs to be evidence about the degree of relationship of the instrument with theoretically related variables. SSMMD (Skinner et al., 2009) assumes that student classroom engagement is related to student need satisfaction and student perceptions of teacher support. More precisely, learners actively engage in their learning activities to the extent that teachers can meet their needs for autonomy, competence, and relatedness (Connell and Wellborn, 1991; Dincer and Dincer, 2012; Noels et al., 2016; Reeve, 2012; Ryan and Deci, 2000). The relationship between student classroom engagement and student need satisfaction was [*r* (383) = 0.571; *ρ* < 0.01], and that between student classroom engagement and student perceptions of teacher support was [*r* (383) = 0.439; *ρ* < 0.01]. The results obtained from this study support the assumption of SSMMD. The above correlation results indicate that the newly developed instrument is valid for measuring student classroom engagement in mathematics and science. The above result is consistent with the result obtained by Skinner et al. (2009).

**Evidence from discriminant validity:** discriminant validity is a crucial component of construct validity, which evaluates the degree to which the newly identified aspects of the student classroom engagement instrument are distinct from one another. The Average Variance Extracted (AVE) and the Fornell-Larcker criteria (1981) were utilized to verify the independence of each dimension. Results from both methodologies indicated that all categories measured distinct characteristics of student classroom engagement. Comparable methodologies were employed by Turan Gürbüz et al. (2020) to get evidence about the discriminant validity of their instrument.

### Instrument reliability

To examine the reliability of the student classroom engagement instrument, internal consistency and test–retest score correlation procedures were used. The results indicated that the full scale has high internal consistency, with a Cronbach’s alpha of 0.914. Reliability results naturally occur between 0 and 1, and the reliability result of this instrument is found to be very high. With respect to the reliability results of the subcomponents of the instrument, cognitive, behavioral, and emotional engagement and disengagement were good, with a Cronbach’s alpha of 0.75. On the basis of the criterion suggested by DeVellis and Thorpe (2021), in terms of reliability values, the newly developed instrument has good internal consistency in measuring student classroom engagement in the science and mathematics classroom context. Similarly, most previously constructed classroom engagement instruments use Cronbach’s alpha to determine the internal consistency of their instrument (Günüç and Kuzu, 2014; Kong et al., 2003; Whitney et al., 2019). In addition, the test–retest reliability result of the instrument [*r* (42) = 0.789, *ρ* < 0.001] indicated that the instrument has to provide consistent results over a period of time.

In the relationship between engagement and disengagement components results were consistent with the study finding of Skinner et al. (2009). The engagement and disengagement components were measure different activates in science and mathematics classroom.

### Measurement invariance

To answer the question of whether the student’s classroom engagement measurement instrument is invariant in terms of gender and grade level, a test of measurement invariance was conducted. This study provides empirical evidence to support measurement invariance by gender and grade level. This suggests that items in the newly developed instrument were viewed and understood similarly by these demographic groupings. Researchers can more appropriately compare groups, such as boys and girls, and those from different SES groups by establishing a measurement of invariance, which ensures that measures of engagement operate similarly across groups.

## Conclusion

The empirical evidence obtained from this study indicates that the newly developed instrument is valid and reliable for measuring student classroom engagement in the Ethiopian context. This study is believed to have made the following contributions: (1) the study has developed and standardized an instrument that addresses the apparent lack of a national-level student classroom engagement instrument for high school students; (2) the results of this study can be used to support courses on instrument development and validation in Ethiopian institutions; and (3) this study contributes to the literature that uses disengagement as a separate dimension to measure student classroom engagement. This allows a researcher to determine how disengagement affects students’ achievement in science and mathematics classrooms.

## Limitations and recommendation

It is important to interpret the findings of this study considering the following limitations.

First, the instrument development processes depended on data gathered from high schools that are found in the southern part of the country. This might be one challenge on the generalizability of the results obtained. Future studies on validating the instrument will need to include data from other geographical locations in addition to the one the present study included. Second, the present study employed self-reported survey instrument which was used as the only method to measure student classroom engagement. Future studies would need to develop student classroom engagement instrument that includes more than one method to get a comprehensive picture about classroom engagement. Third, this instrument assessed classroom engagement only from students’ perspective. Future studies will need to develop classroom engagement instrument including teachers’ view about student classroom engagement. Finally, the sample size that was used for test–retest reliability was very small; this might potentially affect the generalizability of the results. Thus, future studies will need employ adequate sample from different gender and grade levels in order to improve the generalizability results.

## Data Availability

The raw data supporting the conclusions of this article will be made available by the authors, without undue reservation.
