# Supplementary material for: Development and validation of a secondary school classroom engagement instrument in math and science in the Ethiopian context
Source: Front Psychol. 2025 Feb 17;16:1491615. doi: 10.3389/fpsyg.2025.1491615 (PMC11872900; doi:10.3389/fpsyg.2025.1491615)
Supplement: Supplementary file 1 [file Data_Sheet_1.docx]

**Developing and Validating Secondary School Classroom Engagement Instrument in Math and Science in Ethiopian Context**

$\mathbf{Alemayehu Berhanu}^{\boldsymbol{1*}}$**,** $\mathbf{Tesfaye Semela}^{\boldsymbol{2}}$ **,** $\mathbf{Belay Moges}^{\boldsymbol{3}}$

^1^School of Teacher Education, College of Education, Hawassa University, Hawassa, Ethiopia and Dilla University, Department of Psychology

^2^Director of Institute of Policy and Development Research (IPDR), Hawassa University, Main Campus, IPDR Building, Room No. 201, Hawassa, Ethiopia

^3^School of Teacher Education, College of Education, Hawassa University, Hawassa, Ethiopia and Dilla University, Department of Psychology

*Corresponding author* *email*:alex28zed@gmail.com

**Supplementary Materials**

Annex 1-3

**Annex-1**

| Item | Item-total correlation | Item-total correlation with sub components |
| --- | --- | --- |
| 1 | 0.380 | 0.649 |
| 2 | 0.224 | 0.771 |
| 3 | 0.477 | 0.635 |
| 4 | 0.643 | 0.700 |
| 5 | 0.555 | 0.708 |
| 6 | 0.445 | 0.566 |
| 7 | 0.535 | 0.673 |
| 8 | 0.587 | 0.680 |
| 9 | 0.294 | 0.696 |
| 10 | 0.653 | 0.719 |
| 11 | 0.54 | 0.646 |
| 12 | 0.431 | 0.761 |
| 13 | 0.286 | 0.738 |
| 14 | 0.430 | 0.745 |
| 15 | 0.612 | 0.744 |
| 16 | 0.617 | 0.745 |
| 17 | 0.561 | 0.721 |
| 18 | 0.308 | 0.542 |
| 19 | 0.420 | 0.658 |
| 20 | 0.644 | 0.760 |
| 21 | 0.650 | 0.729 |
| 22 | 0.432 | 0.726 |
| 23 | 0.563 | 0.607 |
| 24 | 0.349 | 0.754 |
| 25 | 0.278 | 0.753 |
| 26 | 0.501 | 0.637 |
| 27 | 0.679 | 0.754 |
| 28 | 0.642 | 0.718 |
| 29 | 0.566 | 0.713 |
| 30 | 0.575 | 0.689 |
| 31 | 0.519 | 0.681 |
| 32 | 0.534 | 0.653 |
| 33 | 0.510 | 0.657 |
| 34 | 0.569 | 0.675 |
| 35 | 0.625 | 0.650 |

Annex-2

Student Classroom Engagement Instrument

1. I do irrelevant things when I am supposed to be paying attention in science and math classes
2. I do not try to work very hard at science and math courses
3. I keep trying even if something is hard in science and math courses
4. I make sure to study on a regular basis science and math courses
5. I put effort into learning science/math individually and group
6. I take good notes in class, on readings, and/or on video lectures.
7. I try to do my best regarding my responsibilities in group work on science and math courses
8. In science and math class, I work as hard as I can.
9. When I am in science and math class, I just act as if I am working.
10. When I am in science and math classes, I listen very carefully.
11. I enjoy learning new things about science and math.
12. I do not want to be in science and math classes.
13. I do not care about learning science and math.
14. I often feel down when I am in science and math classes.
15. I am very interested in learning science and mathematics.
16. I am motivated by my desire to learn math and science.
17. I find ways to make science and math course interesting to me.
18. My science and math classwork makes me curious to learn other things
19. I feel bored when I am learning science and math.
20. I feel excited by the learning activities in my science and math classes.
21. When I am in science and math classes, I feel good.
22. When I am working on my science and math classwork, I feel disgusting.
23. I look different ways to solve science and math problems.
24. During science and math classes, I would rather be told the answer than have to master the procedure.
25. When science and math work is hard I only study the easy parts
26. I prepare for science and math courses before going class.
27. I spend enough time and make enough effort to learn science and math
28. When I am studying science and math lessons, I try to connect different topics from course material.
29. I combine ideas from different courses to help complete my science and math assignments.
30. I summarize the material I learn in class or from other course materials.
31. If given, I identify key information from any reading assignment on science and math lessons
32. When I learn a new science lesson, I ask myself questions to make sure I understand what I am learning about.
33. I look for chances to be part of science events that are related to things we are doing in my science class.
34. I look for extra information (books or internet) to learn more about things we do in science classes.
35. If I don’t understand what I read in science and math classes, I go back and read it over again, look it up, discuss it with someone.

**Annex-3**

**Amharic Version of Student Classroom Engagement Instrument Items**

| ተ.ቁ | ጥያቄ | ደረጃ | | | | | |
| --- | --- | --- | --- | --- | --- | --- | --- |
|  |  | 1 | 2 | | 3 | | 4 |
| 1 | **በክፍልውስጥ ሳይንስ እና ሒሳብን ከመከታተል ይልቅ ሌሎች ስራዎችን እሰራለዉ፡፡** |  |  | |  | |  |
| 2 | **በሳይንስ እና ሂሳብ ክፍለ ጊዜያት ጠንክሮ ለመስራት አልሞክርም፡፡** |  |  | |  | |  |
| 3 | **በሳይንስ እና ሂሳብ ክፍለ ጊዜያት አንድ ነገር ምንም እንኳ ን ከባድ ቢሆን መሞከሬን አላቆምም፡፡** |  |  | |  | |  |
| 4 | **ሳይንስና እና ሂሳብ ትምህርቶችን በቋሚነት አጠናለሁ፡፡** |  |  | |  | |  |
| 5 | **የሳይንስ እና ሂሳብ ትምህርቶችን በግል እና በቡድን ለመማር ጥረት አደርጋለሁ፡፡** |  |  | |  | |  |
| 6 | **በትምህርት ጊዜ፣ ሳነብ እና የቪዲዮ ትምህርቶችን ስከታተል ጥሩ ማስታወሻዎችን እይዛለሁ** |  |  | |  | |  |
| 7 | **በሳይንስ እና ሂሳብ ትምህርት የቡድን ስራ ወቅት የተቻለኝን ሁሉ ለማድረግ ጥረት አደርጋለሁ፡፡** |  |  | |  | |  |
| 8 | **በሳንስ እና ሂሳብ ትምህርት የተቻለኝን ያህል ጠንክሬ እሰራለሁ፡፡** |  |  | |  | |  |
| 9 | **በሳይንስ እና ሂሳብ ክፍለ ጊዜየት እየሰራሁ እንደሆነ አስመስላለሁ፡፡** |  |  | |  | |  |
| 10 | **በሳይንስ እና ሂሳብ ክፍለ ጊዜያት በጥንቃቄ አዳምጣለሁ፡፡** |  |  | |  | |  |
| 11 | **ስለሳይንስ እና ሂሳብ አዳዲስ ነገሮችን መማር ያስደስተኛል፡፡** |  |  | |  | |  |
| 12 | **በሳየንስ እና ሂሳብ ክፍለ ጊዜያት ክፍል መግባት አልፈልግም፡፡** |  |  | |  | |  |
| 13 | **ሳይንስ እና ሂሳብ ለመማር ግድ የለኝም፡፡** |  |  | |  | |  |
| 14 | **ለሳይንስ እና ሂሳብ ክፍለ ጊዜያት ስሜቴ ቀዝቃዛ ነው፡፡** |  |  | |  | |  |
| 15 | **ሳይንስ እና ሂሳብ ለመማር ከፍተኛ ፍላጎት አለኝ፡፡** |  |  | |  | |  |
| 16 | **ሳይንስ እና ሒሳብን በራሴ ፍላጎት በመነሳሳት እማራለሁ፡፡** |  |  | |  | |  |
| 17 | **የሳይንስ እና ሂሳብ ፍላጎት እንዲያድርብኝ የተለያዩ ስልቶችን እጠቀማለሁ፡፡** |  |  | |  | |  |
| 18 | **የሳይንስ እና ሂሳብ የክፍልስራዎቼ ሌሎች ነገሮችን ለመማር ገጉት ያጭሩብኛል፡፡** |  |  | |  | |  |
| 19 | **ሳይንስ እና ሂሳብ ስማር ይሰለቸኛል፡፡** |  |  | |  | |  |
| 20 | **የሳይንስ እና ሂሳብ የትምህርት ተግባራት ያነቃቁኛል፡፡** |  |  | |  | |  |
| 21 | **በሳይንስ እና ሂሳብ ክፍል ውስጥ ስሆን ጥሩ ስሜት ይሰማኛል፡፡** |  |  | |  | |  |
| 22 | **የሳይንስ እና ሂሳብ የክፍል ስራዬን ስሰራ ያስጠላኛል፡፡** |  |  | |  | |  |
| 23 | **የሳይንስ እና ሂሳብ ጥቄዎችን ለመፍታት የተለያዩ መንገዶችን እፈልጋለሁ፡፡** |  |  | |  | |  |
| 24 | **በሳይንስ እና ሂሳብ ክፍለጊዜያት ሂደቶቹን ከማወቅ ይልቅ መልሱ ቢነገረኝ እመርጣለሁ፡፡** |  |  | |  | |  |
| 25 | **የሳይንስ እና ሂሳብ ስራ ከባድ ሲሆን ቀላሉን ብቻ አጠናለሁ፡፡** |  |  | |  | |  |
| 26 | **ክፍል ከመግባቴ በፌት ለሳይንስ እና ሂሳብ ትምህርቶች እዘጋጃለሁ፡፡** |  |  | |  | |  |
| 27 | **የሳይንስ እና ሂሳብ ትምህርቶችን ለመማር በቂ ግዜ እመድባለሁ፣ በቂ ጥረት አደርጋለሁ፡፡** |  |  | |  | |  |
| 28 | **የሳይንስ እና ሂሳብ ትምህርቶችን ሳጠና የተለያዩ ርእሶችን እንዲሁም አጋዥ መሳሪያዎችን ለማገናኘት እሞክራለሁ፡:** |  |  | |  | |  |
| 29 | **የሳይንስ እና ሂሳብ ትምህርቶች የተሰጡኝን ስራዎች ለማጠናቀቅ ከተለያዩ ትምህርቶች ሃሳቦችን ለማዋሃድ እሞክራለሁ፡፡** |  |  | |  | |  |
| 30 | **በክፍል ውስጥ የተማርኩትን ወይም ከተለያዩ አጋዦች ያገኘሁትን ፍሬ ነገሩን በአጭሩ ለመረዳት እሞክራለሁ፡፡** |  |  | |  | |  |
| 31 | **በሳይንስ እና ሂሳብ ትምህርቶች ማኝኛውም የንባብ ስራ ቢሰጠኝ ቁልፍ መረጃን መለየት እችላለሁ፡፡** |  |  | |  | |  |
| 32 | **አዲስ የሳይንስ ትምህርት ስማር እየተማርኩ ያለሁትን ነገር መረዳቴን ለማረጋገጥ እራሴን ጥያቄ እጠይቃለሁ፡፡** |  |  | |  | |  |
| 33 | **በክፈል ውስጥ በሳይንስ ክፍለጊዜ ከምናከናውናቸው ነገሮች ጋር ዝምድና ያላቸው የሳይንስ ሆነቶች ላይ ለመሳተፍ አጋጣሚዎችን እፈልጋለሁ፡፡** |  |  | |  | |  |
| 34 | **በሳይንስ ክፍለ ጊዜ ስለምናከናውናቸው ነገሮች ተጨማሪ መረጃ (ከመጻህፈት ወይም ከበይነ መረብ) ለማግኘት እሞክራለሁ፡፡** |  |  | |  | |  |
| 35 | **በሳይንስ እና ሂሳብ ክፍለጊዜያት የማነበውን ካልተረዳሁ ተመልሼ በተደጋጋሚ አነበዋለሁ፣ የተለያዩ መረጃዎችን ለማግኘት እሞክራሉ እንዲሁም ከሌላ ሰው ጋር እወያይበታለሁ፡፡** |  | |  | |  |  |
